# Characterization of the Cry1Ah resistance in Asian corn Borer and its cross-resistance to other *Bacillus thuringiensis* toxins

**DOI:** 10.1038/s41598-017-18586-2

**Published:** 2018-01-10

**Authors:** Muhammad Zeeshan Shabbir, Yudong Quan, Zhenying Wang, Alejandra Bravo, Mario Soberón, Kanglai He

**Affiliations:** 1 State Key Laboratory for Biology of Plant Diseases and Insect Pests, Institute of Plant Protection, Chinese Academy of Agricultural Sciences, Beijing, 100193 People’s Republic of China; 2 Departamento de Microbiología Molecular, Instituto de Biotecnología, Universidad Nacional Autónoma de México, Apdo. Postal 510–3, Cuernavaca, 62250 Morelos Mexico

## Abstract

Transgenic crops producing insecticidal proteins are effective to manage lepidopteran pests. Development of insect-resistance is the major threat to *Bacillus thuringiensis* (Bt) crops such as Cry1Ah-Maize. Laboratory selection with Bt-Cry1Ah toxin incorporated in artificial diet, during 48 generations of Asian corn borer (ACB) *Ostrinia furnacalis* produced 200-fold resistance. This resistant colony ACB-AhR readily consumed and survived on Cry1Ah-expressing Bt-maize. Cross-resistance analysis showed high cross-resistance to Cry1F (464-fold), moderate cross-resistance to Cry1Ab (28.38-fold), Cry1Ac (22.11-fold) and no cross-resistance to Cry1Ie toxin. This ACB-AhR cross-resistant phenotype is different from ACB-Cry1Fa resistant population that showed no cross resistance to Cry1Ah, suggesting that different mechanisms of resistance were selected in these two populations. Bioassays of reciprocal F_1_ crosses-progeny suggested autosomal inheritance of Cry1Ah resistance with no maternal effects. The dominance of resistance increased as concentration decreased. In Cry1Ah-maize tissues the progeny of reciprocal F_1_ crosses behaved as functionally recessive. Progenies analysis from backcrosses (F_1 × _resistant strain) suggested polygenic contribution to Cry1Ah- resistance in ACB-AhR. The use of multiple toxins is an imperative factor for delaying evolution of resistance to Cry1Ah-corn in ACB. However, the fact that ACB-AhR showed cross resistance to Cry1Fa indicates that selection of toxins for pyramided plants should be carefully done.

## Introduction

To improve insect pest management, genetic modified crop plants such as cotton and corn based on insecticidal proteins from *Bacillus thuringiensis* (Bt) are now the most widely used strategies^1^. Since failures of synthetic chemicals, microbial insecticides derived from Bt have proven ample potential for pest management^2^. Insecticidal proteins are extensively used both in sprays formulations and transgenic crops to control lepidopteran pests having negligible effects on non-target pests^3–5^. These Bt insecticidal proteins are valuable as they can bring season long protection against lepidopteran pests^6^.

Since worldwide commercialization of transgenic Bt crops in 1996, cry genes that codify for insecticidal proteins such as Cry1Ab, Cry1Ac, Cry1F, Cry1Ah, and Cry3Bb1 have been expressed in corn, cotton and soybean and grown extensively in several countries^7^. However, the efficacy of transgenic crops can be reduced by the continuous use of single trait products. Insect resistance is the major hazard to the enduring success of Bt crops^8^. Bt resistance in field conditions has been reported in different insect pests including *Ostrinia nubilalis* ^9^, *Plutella xylostella* ^10^, *Trichoplusia ni* ^11^, *Spodoptera frugiperda* ^12^, *Busseola fusca* ^13^, *Diabrotica virgifera virgifera* ^14^ and *Pectinophora gossypiella* ^15^. In order to delay resistance evolution, implementation of suitable resistance management practices is necessary. The application of high-dose/refuge strategy and pyramiding of two or more toxins with different mode of action have been the foremost strategies used worldwide to delay evolution of resistance in different pests toward Bt crops^16–18^. The theory underlying the refuge strategy implies the planting of non-Bt host plants in close vicinity to Bt crops to assure that the rare resistant individuals selected on Bt crops will mate with susceptible individuals from nearby refuges of host plants without Bt toxins therefore generating heterozygous progenies that should be sensitive to the high dose concentration of the Bt toxin expressed in the transgenic plants, implying recessive inheritance^19–22^. Multiple components are predicted to favor the success of the refuge strategy: recessive inheritance of resistance, low initial resistance allele frequency, pest movements and mating patterns, fitness costs and incomplete resistance^19,23^. Moreover, the success of the strategy based on applying rotations of different toxins or toxin mixtures works best if the inheritance of resistance to each toxin is recessive.

Transgenic corn expressing the activated 65 kDa Cry1Ah was developed by Origen Seeds Ltd to target Asian corn borer (ACB). Knowledge of the genetic basis of resistance to Cry1Ah toxin is imperative for designing and refining managing tactics to minimize evolution of resistance in this pest. The present study offers insights into the mechanisms linked to resistance evolution. We describe here the results of the experiments that analyzed the mode of inheritance for Cry1Ah resistance in a laboratory selected strain resistant to Cry1Ah (ACB–AhR), we evaluated maternal effects, sex linkage and effective dominance. In addition, backcrosses were performed to estimate the number of loci affecting the inheritance. Also, the assumption of functional recessive resistance to Cry1Ah toxins was measured by determining the survival of Cry1Ah-resistant, susceptible parental strains and the F_1_ progenies from reciprocal crosses in experiments with Bt corn plants tissues and non-Bt plant in laboratory bioassays. Finally, cross resistance patterns among different Bt toxins were also analyzed. The implications of this study will be helpful for the future managing of ACB resistance to transgenic Cry1Ah-maize.

## Results

### Selection of ACB for Cry1Ah resistance

Bioassays using Cry1Ah toxin and Bt maize tissues revealed that ACB-AhR strain developed a very high level of resistance to Cry1Ah toxin after 48 generations of selection. The diet bioassays validated that larvae from the resistant strain had a higher level of resistance to Cry1Ah toxin compared with larvae from the susceptible strain. The LC_50_ values for the susceptible (ACB-BtS) and Cry1Ah-selected colonies (ACB-AhR) were significantly different showing LC_50_ values of 0.33 and 63.91 μg/g Cry1Ah toxin, respectively, (Table 1). The differences in LC_50_ values between ACB-BtS and ACB-AhR strains and the resulting resistance ratio of more than 193.67-fold validates that resistance to Cry1Ah toxin is attainable for this species.

**Table 1:** Susceptibility of ACB-AhR and ACB-BtS of *O*. *furnacalis* to 5 Bt toxins.

Bt toxin	Strain	n^a^	LC_50_ (95% FL) (µg/g)	Slope ± SE	χ^2^	df (χ^2^)	RR^b^
Cry1Ah	ACB-BtS^c^	768	0.33 (0.27–0.39)	1.667 ± 0.120	5.11	12	—
	ACB-AhR^d^	1296	63.91 (54.77–74.38)	1.292 ± 0.065	13.30	22	193.67
Cry1Ab	ACB-BtS	336	0.21 (0.16–0.27)	1.758 ± 0.192	1.82	5	—
	ACB-AhR	336	5.96 (4.86–7.25)	1.929 ± 0.173	2.36	5	28.38
Cry1Ac	ACB-BtS	336	0.28 (0.19–0.38)	1.774 ± 0.181	5.56	5	—
	ACB-AhR	576	6.19 (5.05–7.42)	1.488 ± 0.125	7.31	10	22.11
Cry1Ie	ACB-BtS	336	7.53 (6.17–9.14)	1.975 ± 0.174	3.76	5	—
	ACB-AhR	672	7.29 (6.31–8.43)	1.826 ± 0.117	2.29	12	0.97
Cry1F	ACB-BtS	336	0.39 (0.25–0.53)	1.313 ± 0.174	1.66	5	—
	ACB-AhR	672	181.02 (150.84–222.54)	1.457 ± 0.112	2.77	12	464.10

^a^n, number of larvae tested.

^b^RR, resistance ratio = LC_50_ of particular strain or cross divided by LC_50_ of susceptible strain.

^c^ACB-BtS is susceptible strain.

^d^ACB-AhR is the resistant strain of *Ostrinia furnacalis*.

### Cross-resistance

The LC_50_ values for Cry1Ab and Cry1Ac were significantly higher in the ACB-AhR strain compared to the ACB-BtS strain, i.e. bioassays with Cry1Ac led to a 22.11-fold resistance, indicating that the selected ACB-AhR strain is slightly cross-resistance to Cry1Ac. Similarly, a 28.38-fold resistance was observed when exposed to Cry1Ab toxins (Table 1). The highest level of cross-resistance was detected with Cry1F with a resistance ratio of up to 464 fold, whilst no cross-resistance was observed with Cry1Ie (Table 1). These data indicate that there is no cross-resistance between Cry1Ie and Cry1Ah in ACB-AhR strain.

### Survival on plant tissues

Neonate larvae of ACB-AhR, ACB-BtS strains and their F_1_ progeny displayed significantly lower rate of feeding on Bt maize tissues than on non-Bt maize tissues. The survival rates of ACB-AhR strain were significantly greater than ACB-BtS strain and F_1_ progenies when larvae were fed on Cry1Ah-expressing maize leaf tissue after 7 days (Table 2). In addition, ACB-AhR strain exhibited less survival on Bt kernel followed by Bt leaves, highest survival of resistant strain was perceived on silk tissues (Table 2). In contrast, in case of non-Bt leaves these strains did not differ significantly (*F*_3, 8_ = 12.3; *P* = 0.0023). ACB-AhR strain showed more survival rate than susceptible strain when fed on non-Bt silk tissues (*F*_3, 8_ = 3.52; *P* = 0.0689). The larvae of all strains significantly differed when they were fed on kernel tissue of non-Bt maize (*F*_3, 8_ = 25.2, *P* = 0.0002) (Table 2).

**Table 2:** Survival of ACB-AhR, ACB-BtS strains of *Ostrinia furnacalis* and F_1_ progenies fed on Bt and non-Bt maize tissues.

Insect Strains	Leaf (%)		Silk (%)		Kernel (%)	
	Cry1Ah-corn	Non-Bt	Cry1Ah-corn	Non-Bt	Cry1Ah-corn	Non-Bt
ACB-AhR	6.3 ± 1.2 a	80.6 ± 2.5 a*	60.0 ± 2.9 a	86.7 ± 3.3 a*	2.7 ± 0.6 a	79.3 ± 2.4 a*
ACB-BtS	0.7 ± 0.7 b	75.0 ± 3.2 a*	0.0	80.0 ± 2.5 ab*	0.0	60.0 ± 5.2 b*
S♂ × R♀	1.4 ± 0.7 b	78.5 ± 1.8 a*	3.3 ± 0.8 b	84.2 ± 1.7 a*	1.3 ± 0.3 b	52.7 ± 1.3 bc*
R♂ × S♀	0.7 ± 0.7 b	59.7 ± 3.0 b*	0.0	68.3 ± 7.4 b*	0.0	49.2 ± 1.2 c*

Data are means ± SE.

The means within column followed with same letter are not significantly different (*P* < 0.05) and the “*” indicate that Cry1Ah-corn and non-Bt corn are significantly different according to Fisher’s Protect LSD test.

### Quantification of Cry1Ah toxin

**Figure 1:**
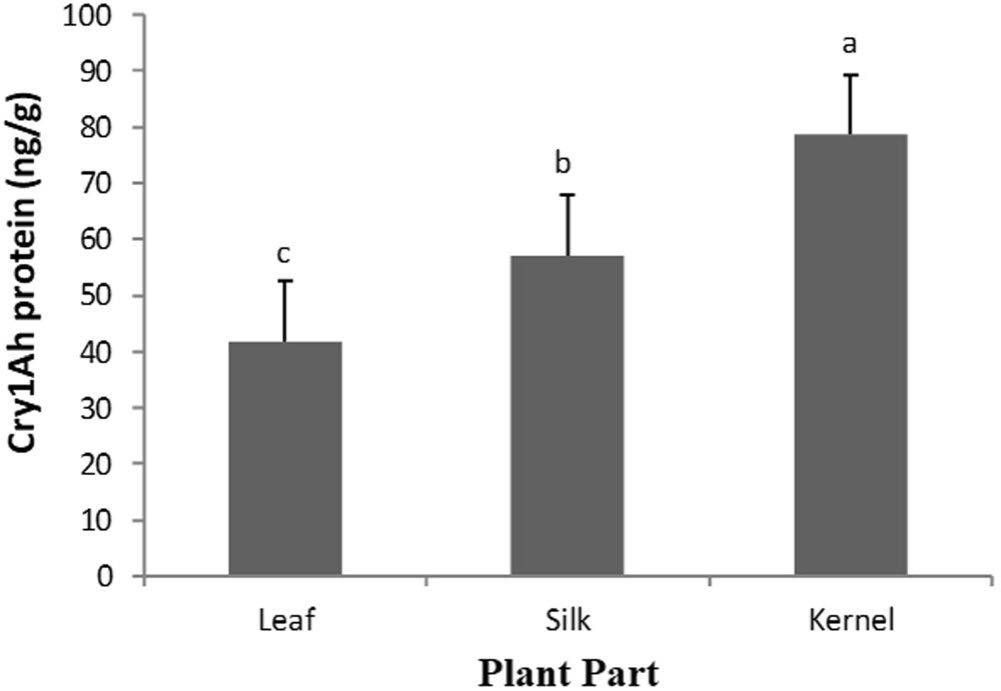
Quantification of Cry1Ah toxin (ng/g) in Cry1Ah maize leaves, silk and kernel tissue. Means ± SE followed by different letters are significantly different (*P* < 0.05) according to Fisher’s Protect LSD test. The concentration level of Cry1Ah toxin increased through the leaf to kernel tissue.

Cry1Ah concentrations were determined in leaves, silk and kernels tissues of the Cry1Ah- maize. Kernel showed the highest concentration of Cry1Ah (Fig. 1). The concentration of Cry1Ah toxin in these plant tissues was 41.84, 57.10 and 78.66 ng/g dry weight, respectively (*F*_2, 29_ = 28.3; *P* < 0.05).

### Maternal effects and sex linkage

The maternal influence and the sex-linked nature of the resistance were examined by comparing observed larval concentration responses of the F_1_ progeny. The LC_50_ for the F_1_ progeny from reciprocal crosses to Cry1Ah was significantly greater than the LC_50_ for the susceptible parental strain and significantly less than the LC_50_ for the resistant parental strain (Table 3). Likewise, the mean slope of the concentration-mortality line did not differ between the reciprocal crosses. Thus, inheritance was autosomal; neither maternal effects nor sex-linkage were evident.

**Table 3:** Responses of F_1_ progenies from reciprocal crosses between resistant and susceptible strains of *Ostrinia furnacalis* to Cry1Ah toxin.

Strain	n^a^	LC_50_ (95% FL) (µg/g)	Slope ± SE	χ^2^	χ^2^ (df)	RR^b^
ACB-BtS^c^	768	0.45 (0.37–0.59)	1.776 ± 0.124	12.32	12	—
R♂ × S♀^d^	1152	2.89 (2.46–3.39)	1.340 ± 0.080	7.11	19	6.42
S♂ × R♀^e^	1152	3.05 (2.60–3.59)	1.337 ± 0.079	7.63	19	6.78

^a^n, number of larvae tested.

^b^RR, resistance ratio = LC_50_ of strain or cross divided by LC_50_ of susceptible strain.

^c^ACB-BtS is susceptible strain of *Ostrinia furnacalis*.

^d^Progeny of mass cross between resistant male and susceptible female.

^e^Progeny of mass cross between susceptible male and resistant female.

### Estimation of the degree of dominance

Dominance estimations at different concentrations of Cry1Ah toxin revealed that the resistance was dominant in low concentration treatment but decreased if the concentration increased. The level of dominance is obtained by the calculation of effective dominance at different toxin concentrations, *h* varied with concentration, from dominant inheritance at low concentrations to recessive inheritance at high concentrations. For example, results presented partially dominance at 0.5 μg/g (*h* = 0.79), and declined to incomplete-recessive by treatment concentrations of 50.0 μg/g (*h* = 0.4) and complete recessive 250 μg/g (*h* = 0) (Table 4).

**Table 4:** Effective of dominance (*h*) of resistance to Cry1Ah-selected Asian corn Borer.

Concentration (μg/g)	Strains	Survival (%)	Fitness^a^	*h* ^b^
0.5	ACB-BtS	38.5	0.39	0.79
	ACB-AhR	96.9	1.00	
	F1	84.4	0.87	
2.5	ACB-BtS	12.5	0.13	0.65
	ACB-AhR	95.8	1.00	
	F1	66.7	0.69	
5.0	ACB-BtS	5.2	0.55	0.60
	ACB-AhR	93.8	1.00	
	F1	58.3	0.62	
12.5	ACB-BtS	0.0	0	0.57
	ACB-AhR	81.3	1.00	
	F1	46.9	0.58	
50.0	ACB-BtS	0.0	0	0.35
	ACB-AhR	50.0	1.00	
	F1	17.7	0.35	
250.0	ACB-BtS	0.0	0	0
	ACB-AhR	28.1	1	
	F1	0.0	0	

^a^Fitness of the susceptible parent and the reciprocal cross was estimated from the survival rate of the larvae at a specific treatment concentration divided by the survival rate of the resistant parent at the same concentration.

^b^ *h* = (ωRS − ωSS)/(ωRR − ωSS), where ωRS is the fitness of the heterozygous offspring, ωSS is the fitness homozygous susceptible parent, ωRR is the fitness of homozygous resistant parent. *h* can vary from 0 (completely recessive resistance) to 1 (complete dominant resistance).

Plant tissue bioassays showed that the *h* values were 0.13, 0.06 and 0 for Cry1Ah maize leaves, silks and kernel, respectively (Table 5), which indicated that the resistance was functionally recessive at leaves stage of maize plant development. At the silking stage of the plant, the dominance was virtually recessive but it was completely recessive at the kernel stage of the plant development.

**Table 5:** Effective dominance values (*h*) of Cry1Ah resistance in ACB-AhR strain of the *Ostrinia furnacalis* based on Cry1Ah maize plant tissues bioassays.

Plant Tissue	Strains	Survival (%)	Fitness^a^	*h* ^b^
Leaves	ACB-BtS	0.7	0.11	0.13
	ACB-AhR	6.3	1	
	F1	1.4	0.23	
Silk	ACB-BtS	0.0	0	0.06
	ACB-AhR	60.0	1	
	F1	3.3	0.06	
Kernel	ACB-BtS	0.0	0	0
	ACB-AhR	2.0	1	
	F1	0.0	0.50	

^a^Fitness of the susceptible parent and the reciprocal cross was estimated from the survival rate of the larvae at a specific treatment concentration divided by the survival rate of the resistant parent at the same concentration.

^b^ *h* can vary from 0 (completely recessive resistance) to 1 (complete dominant resistance) (see Materials and Methods).

### Number of loci influencing resistance

The F_1_ population was backcrossed with ACB-AhR and tested for goodness-of-fit to a monofactorial model. The direct tests use chi-square values calculated from the observed and expected mortalities of the backcross population. The pattern of response was not consistent with a monofactorial model (∑χ^2^ = 43.61 > ($\sum {\chi }_{0.05}^{2}$ = 3.84 (df = 1) (Table 6). The same response was observed in all backcross interactions showing that inheritance of resistance to Cry1Ah in Asian corn borer ACB-AhR strain is polygenic.

**Table 6:** Direct test for deviation between observed and expected mortality for a monogenic model (df = 1).

Concentration (μg/g)	Actual mortality (%)	Expected mortality (%)	χ^2^	*P*
RR♀ × F_1_♂ (SS♀ × RR♂)	—	—	—	—
0.5	7.3	8.3	0.14	0.712
2.5	39.6	28.1	6.23	0.013^a^
5.0	48.9	35.4	7.69	0.006^a^
12.5	63.5	47.9	9.39	0.002^a^
25.0	81.2	58.7	20.16	>0.001^a^
$\sum {{\rm{\chi }}}^{2}$			43.61	
F_1_♀ (SS♀ × RR♂) × RR♂				
0.5	7.3	8.3	0.13	0.712
2.5	19.8	28.1	3.29	0.069
5.0	44.8	35.4	3.68	0.055
12.5	59.4	47.9	5.05	0.025^a^
25.0	75.0	58.7	10.54	0.001^a^
$\sum {{\rm{\chi }}}^{2}$			22.69	
RR♀ × F_1_♂ (RR♀ × SS♂)				
0.5	7.2	8.3	0.13	0.712
2.5	26.0	24.3	0.15	0.692
5.0	42.7	35.8	2.01	0.156
12.5	64.6	47.9	10.68	0.001^a^
25.0	86.4	57.9	31.94	>0.001^a^
62.5	95.8	79.5	15.69	>0.001^a^
$\sum {{\rm{\chi }}}^{2}$			60.63	
F_1_♀ (RR♀ × SS♂) × RR♂				
0.5	7.3	8.3	0.13	0.712
2.5	23.9	24.3	0.06	0.937
5.0	38.5	35.8	0.32	0.570
12.5	58.3	47.9	4.17	0.041^a^
25.0	81.2	57.9	21.32	>0.001^a^
62.5	94.8	79.5	13.75	>0.001^a^
$\sum {{\rm{\chi }}}^{2}$			39.64	

## Discussion

Bt maize, specifically the first generation of biotech Bt maize hybrids target both *O*. *nubilalis* and *O*. *furnacalis*. A few strains of *O*. *nubilalis* have developed resistance to the expressed protein in different events of Bt maize, Cry1Ac, Cry1Ab or Cry1F in laboratory-selection experiments^24–27^. In this work we showed that the selection of *O*. *furnacalis* with Cry1Ah resulted in a high level of resistance to this toxin (200-fold for ACB-AhR). We showed that ACB-AhR could feed and survive on leaf, silk and kernel tissues of Bt-maize, although survival rates were low on leaves and kernels (Table 2). The low survival on Cry1Ah-leaves of ACB-AhR was unexpected since this tissue showed the lower Cry1Ah concentration (Fig. 1). It could be possible that other plant molecules present in leaves and kernels of Cry1Ah-maize contribute to the low survival rate observed in these tissues. In agreement with this, both ACB-BtS and ACB-AhR showed higher survival rates on non-Bt silk than on non-Bt leaves or kernels (Table 2). In earlier findings, Cry1F-selected strain (ACB-FR) established a high level of resistance (1700-fod) and the larvae presented potential to survive and consume Cry1F toxin^27^. Four strains of *O*. *nubilalis* that were exposed to Cry1Ab toxin for 10 generations developed low level of the resistance (2.0- to 10-fold) and caused reduced susceptibility to other toxins to which these selected strains were not exposed^26^. However, high level of resistance to Cry1Ac with a peak of 162-fold resistance was observed in a Minnesota strain of *O*. *nubilalis* after only eight generations of selection^24^.

Several factors are involved in resistance levels among strains. However, resistance levels are linked to the number of generations selected, concentrations of toxins consumed, selective agents itself, bioassay procedures and susceptibility among unselected strains^28–30^. In the present study, the levels of resistance to Cry1Ah in laboratory-selected strains resulted in about 200-fold resistance to purified Cry1Ah toxin under moderate selection pressure. These are the highest levels of the resistance to a Cry1Ah toxin ever documented for *O*. *furnacalis*. Previously, different *O*. *furnacalis* resistant strains to different Cry1 toxins were selected and characterized, including 106-fold resistance to Cry1Ab^28^, 40-fold and 113-fold resistance to Cry1Ab and Cry1Ac respectively^31^, or 1700-fold resistance to Cry1F after 49 generations of selection^32^. Factors contributing in increased tolerance to Bt crops may be the time exposure to Bt crops, resistance genes and genetic background of different populations^33^.

The results from the analysis of F_1_ progenies from reciprocal crosses performed between resistant and susceptible strains suggest that resistance to Cry1Ah was autosomally inherited with no maternal effects. The values of the present study are consistent with nearly all previous findings with Bt resistance including resistance to Cry1F in *O*. *furnacalis* ^32^, or to Cry1Ab and Cry1Ac^31^, Greenhouse-Derived Strain of *Trichoplusia ni* to Cry1Ac and *B*. *thuringiensis* subsp. *kurstaki*,^11,34^, *Helicoverpa armigera* to Cry1Ac^35^ and resistance to Cry1F maize in a strain of *S*. *frugiperda* ^36^. Usually, resistance to Bt toxins behave in agreement with autosomal mode of inheritance. In contrast, inheritance of Cry1Ab resistance in a field-derived strain of *O*. *nubilalis* and resistance to Cry1Ac in *P*. *xylostella* in leaf dips bioassay had proven maternal influence on the survival of F_1_ progenies^9,37^. Also, sex linkage phenomenon was described to inheritance pattern of resistance to Cry3Bb1 in *Diabrotica virgifera virgifera* ^38^. These dissimilarities in results are possibly related to use of different insects species.

The spectrum of cross-resistance occurs when the Bt toxins demonstrate similarities in their mechanisms of actions^39,40^. In the present study, selection for Cry1Ah resistance has resulted in slight cross-resistance to Cry1Ab and Cry1Ac with resistance ratios of 28.38 and 22.11-fold, respectively. Cross-resistance of Cry1Ah was not unexpected for Cry1Ab and Cry1Ac because the toxic fragment of Cry1Ah has 72 and 82% similarity to those of Cry1Ab and Cry1Ac, respectively^41^. Similarly, in *O*. *furnacalis*, a Cry1Ab-selected strain has indicated cross-resistance to Cry1Ah^28^. The highest level of cross-resistance was with Cry1F toxin (464-fold). This high level of cross-resistance between Cry1Ah and Cry1F indicates that these toxins shared similar mechanisms of action and sequence similarities. These results varied from the previous study in resistance of *O*. *furnacalis* to Cry1F that showed no cross-resistance to Cry1Ah^32^, suggesting that in these two populations a different mechanism of resistance was selected. The different cross-resistance patterns of these two populations could be due to the different protein used for the selection procedure (Cry1Fa vs. Cry1Ah). Our results suggest that in ACB-AhR resistant population a step shared between Cry1Ah and Cry1Fa toxins was affected, while in the ACB-FR resistant population a step involved in Cry1Fa action but not in Cry1Ah was affected. The presence of multiple binding sites shared by different Cry toxins have been documented before, for example the binding competition assays previously performed in *Diatraea saccharalis* that showed that Cry1Ac and Cry1Ab share binding sites, while Cry1Fa shared binding sites with Cry1Ab but not with Cry1Ac toxin^42^. These results indicate that in *D*. *saccharalis* Cry1Ab toxin may have two binding sites, one that is shared with Cry1Ac and the other shared with Cry1Fa^42^. Our data suggest that in the two *O*. *furnacalis* populations, ACB-AhR and ACB-FR, different Cry1Ah binding sites were affected, one shared with Cry1F and other not shared with Cry1F, explaining the differences observed in cross-resistance in these populations. Our results indicate that the lack of cross-resistance in a Cry resistant population does not necessary means that cross-resistance to other Cry toxins could not be selected when the insects were exposed to a different toxins. In some lepidopteran insect cross-resistance pattern of Cry1F to other Bt toxins has been defined. In *O*. *nubilalis* a strain selected for Cry1F resistance (3000-fold resistance), showed no cross-resistance to Cry1Ab or Cry9C, and a low cross-resistance to Cry1Ac (7-fold)^40^. Interestingly, no cross-resistance was observed to Cry1Ie. The lack of cross-resistance to Cry1Ie to other Cry toxins agree with the previous binding analysis in *O*. *furnacalis* ^28,31,32^. This lack of cross-resistance may be due to binding of the secondary toxins on different binding receptors on brush border membrane vesicles (BBMV). When cross-resistance is not observed, binding studies have shown that the two toxins interact with different binding sites^43^.

The LC_50_ values of F_1_ progenies were intermediate between the LC_50_ values of resistant and susceptible parental strains. The *h* varied from 0 to 0.79 depending on the concentrations of the toxin, with resistance fully recessive at high concentration and almost partially dominant as concentration decreased. In previous studies similar findings have been reported in other insects^9,37^. Analysis of dominance values on plant tissues of Bt-maize showed that the Cry1Ah-resistance was partially recessive on leaves, nearly recessive on silk stage, but completely recessive on Cry1Ah maize kernel. These results show that high dose/refuge strategy is likely to be successful assuring high dose of Cry1Ah in transgenic crop. Dominance is dependent on several factors including the resistance level, toxins and specific strains^30^. High variability in the degree of dominance has been observed in different population^9,44^ and resistance is incompletely recessive in *S*. *frugiperda* even with low dose of Cry1F maize^45^. In contrast, some studies indicated that dominance decreased with higher levels of resistance in the selected strains^29^.

Toxicity assays of backcrosses (RS × RR) showed that the inheritance of resistance to Cry1Ah toxin in ACB-AhR was controlled by more than one locus. The Lande’s method to estimate number of loci involved in resistance is based on the expected mortality of offspring from the RS × RR backcross at each dose of toxin. This test of monogenic model analyzing the response of backcross progenies provided confirmation that more than one locus contributes for Cry1Ah resistance in ACB-AhR. The null hypothesis tested in the standard backcross method is that resistance is controlled by one locus with two alleles^46^. If the resistance had been controlled entirely by one locus with two alleles, the sole RR allele would have been fixed in several generations of selection and further increase in resistance would not have occurred^47^. The genetic basis for resistance to Bt toxins appears to be different in different insect species and different selection regimes. Resistance to Cry1Ac was controlled primarily by one or a few major loci in a field-derived strain of pink bollworm^47^. Furthermore, resistance to Cry1Ab resulted in polygenic control in Cry1Ab-selected Asian corn borer (ACB-AbR), but in Cry1Ac- selected Asian corn borer (ACB-AcR) resistance was controlled by a single locus^31^. A field-derived strain of diamondback moth fit best to polygenic inheritance to Cry1Ac^48^. Resistance to Cry1F in a population of *O*. *nubilalis*, the best fit happened with a single locus or a set of tightly linked loci^49^, and resistance to Cry1Ab in a population from the Philippines revealed contributions from two different genes^50^. Besides, resistance to Cry1Ac toxin in *H*. *armigera* initially was monogenic, but showed better fit to polygenic control as resistance increased^29^.

ACB-AhR laboratory-selected strain showed increased survival on Cry1Ah expressing leaf and silk tissues, although the surviving level was low in kernel tissue of transgenic maize. The Cry1Ah resistance was primarily autosomal, no maternal effects and under polygenic control. Dominance varies depending on toxin concentrations but resistance to Cry1Ah in ACB-AhR was functionally recessive in Bt-maize. These results should be useful in dealing the development of resistance management strategies, and the mechanism of inheritance and cross-resistance will be valuable to minimize the negative effects of cross-resistance on the durability of Bt toxins. Pyramided Bt crops that express two or more toxins that protect against same insect pest is considered a key tactic deployed to delay evolution of pest resistance to succeed in reducing refuges. One of the important components favoring the durability of pyramided Bt crops is that these plants express Cry proteins that lack cross-resistance. Our results show that ACB–AhR has high cross resistance with Cry1Fa toxin, indicating that careful studies should be done to determine cross-resistance since the absence of cross-resistance between two toxins derived from a resistant strain selected against only one-toxin could be not enough to make a decision to develop pyramided Bt crops. This should be taken in account for future development of genetically modified pyramided Bt crops.

## Materials and Methods

### Ethics statement

For insect collection in maize field, no specific permits were required by authorities.

None of the species used in this study are endangered or protected.

### Insect strains

Susceptible and resistant strains of *O*. *furnacalis* used in the present study were obtained from laboratory colonies. A susceptible strain of Asian corn borer (ACB-BtS) was originally collected from corn fields in Liaoning Province. This population was established in the laboratory by rearing on artificial diet without exposure to insecticides for 23 generations, at the Institute of Plant Protection (IPP), Chinese Academy of Agricultural Sciences (CAAS), Beijing. A Cry1Ah-resistant strain (ACB-AhR) was established by 90 pairs of male and female larvae, collected from the fields in the Shaanxi province located in summer maize region of central China. The resulting offspring were used to establish a laboratory strain that was maintained using standard rearing techniques under selection pressure with increasing concentrations of Cry1Ah protein incorporated into artificial diet for several generations. The concentrations initially was 0.05 μg/g (Cry1Ah toxin/diet), and was increased to 0.1 μg/g in the 2^nd^ generation, 0.2 μg/g in the 3^rd^ generation, 0.4 μg/g in the 4^th^ generation, 0.8 μg/g in the 5^th^–7^th^ generation, 2.5 μg/g in the 8^th^–10^th^ generation, 4.0 μg/g in the 11^th^–13^th^ generation, and 6.0 μg/g in the 14^th^–23^th^ generation. The resistance characteristics were tested at the 48^th^ generation. Larvae were reared at 27 ± 1 °C, 70–80% relative humidity (RH), with a photoperiod of 16:8 h light: dark (L:D).

### Bt toxins

Cry1Ah expressed in Bt subsp. *kurstaki* strain Cry1Ah (HD-73) was trypsin-activated and used for selection and bioassays. Trypsin-activated Cry1Ab, Cry1Ac and Cry1F (98% pure protein), were produced by Marianne P. Carey, Case Western Reserve University, USA. Cry1Ie expressed as a recombinant protein in *E*. *coli* ^51^, was provided by the Biotechnology Group of Institute of Plant Protection, Chinese Academy of Agricultural Sciences. Cry1Ie protoxin was purified via NiNTA affinity chromatography and Superdex- 75 size-exclusion chromatography. These samples were analyzed by SDS-PAGE (8%), and the protein concentrations were determined (Universal Hood II, Bio-Rad, USA) using bovine serum albumin (BSA) as a standard. Test solutions of toxins were freshly prepared in distilled water with sodium carbonate in 50 mM sodium carbonate buffer pH_10_.

### Quantification of Cry1Ah toxin expressed in Bt maize tissues

To determine the Bt proteins concentration in the corn leaves, silk and kernel tissues, 10 samples from each tissue were taken randomly. The leaf, silk and kernel tissues were diluted at a rate of 1:10 (milligram sample: microliter PBST buffer) and fully ground by mortar and pestle. The concentration of Cry1Ah in maize leaves, silks, and kernels was confirmed by enzyme-linked immunosorbent assay (ELISA) with Cry1Ah detection kits as per the protocol of manufacturer (Shanghai MLBIO Biotechnology Co. Ltd.).

### Plant tissue bioassays

ACB neonate larvae were tested on the leaves, silks and kernels of transgenic maize (Bt-21) expressing Cry1Ah protein and a non-Bt control. Resistance of ACB-AhR to Bt transgenic maize was evaluated with leaves, silks and kernel to homozygous parental strains (ACB-BtS & ACB-AhR and F_1_ progenies). Hybrid F_1_ progenies were derived from reciprocal crosses between susceptible and resistant strains. To generate the F_1_ progeny, corn borer pupae were separated by gender, and 150 Cry1Ah-selected females were pooled with 150 control males, and 150 control females were pooled with 150 Cry1Ah-selected males in mating cages. The leaves were cut into small pieces with a pair of scissors and placed in individual wells of a 24-well rearing plates. One neonate larva was infested in each well, and then covered with a piece of moistened filter paper and lid. Plates were then incubated at 27 ± 1 °C, 70% RH and a photoperiod of 16:8 (L:D). The number of surviving larvae was recorded either daily or every 2 days, and fresh tissue was provided when required. The bioassays were evaluated in terms of insect survival. Each strain was assayed with 120 larvae and replicated three times. Silk tissue from 10 plants of Bt and non-Bt were collected and were placed into a plastic container with a disc of wet filter paper at bottom. Ten larvae (<12 h after hatching) were placed inside each container using a fine brush, and then covered with a piece of moistened filter paper and lid. Kernels were collected from field and were fast frozen with liquid nitrogen. 15–20 grains of kernel from Bt and non-Bt plants were placed into a container and were infested with 10 larvae. All rearing trays and containers were kept in an incubator 27 ± 1 °C with a photoperiod of 16:8 h (L:D) and 80% RH. The number of surviving larvae were recorded either daily or every 2 days, and fresh tissues were provided when necessary.

### Diet bioassay

We used survival bioassays to evaluate susceptibility to Cry1Ah of the susceptible strain, the resistant strain, and progeny of mass crosses. Bt test solutions were serial diluted in water; we tested 6 to 9 concentrations from 0.2–12.5 μg/g (toxin/diet) for ACB-BtS and 5–800 μg/g (toxin/diet) for ACB-AhR. Dilutions were added to an agar-free semi-artificial diet to form a testing medium^52^, which was then distributed into individual cells of 48-well trays. One neonate ACB larvae (<12 h after hatching) was placed in each well and plates were concealed with adhesive plate sleeves. The larval trays were placed in a climate controlled room 27 ± 1 °C with a photoperiod of 16:8 h (L:D) and 80% RH for 7 days and checked for survival. Those larvae that were visibly moving or that move when poked with a pin were considered surviving larvae. Bioassays were replicated three times in each experiment including a negative control that did not contain any Bt toxin. Cross-resistance bioassays with Cry1Ab, Cry1Ac, Cry1F and Cry1Ie were conducted using the methods defined above.

### Inheritance experiments

To evaluate sex linkage and dominance the influence of sex on Cry1Ah inheritance was tested by bioassays of F_1_ progeny from reciprocal mass crosses between resistant and susceptible strains. The power of indirect tests for modes of inheritance is higher when the backcross progeny are originated from crosses between F_1_ progeny and the parental strain which is more dissimilar in susceptibility to the toxin^46^. In the reciprocal mass cross between resistant and susceptible strains, 100 resistant female pupae were pooled with 100 susceptible male pupae in one cross. In another reciprocal cross, we pooled 100 resistant male pupae with 100 susceptible female pupae. The estimation of number of genes involved in inheritance of Cry1Ah resistance was carried in bioassays of backcross progenies. Males and females of each F_1_ were backcrossed to resistant parental strains to produce four backcross populations and tested for susceptibility as described above.

### Statistical analysis

For bioassays with multiple concentrations, probit regression using POLO-PC (LeOra Software 1987) was used to estimate the LC_50_ with 95% fiducial limits (FL), as well as the slopes of the concentration-mortality lines and their standard errors, Chi-square (χ^2^) values and resistance ratio (RR). Resistance ratios were calculated by dividing the LC_50_ for a particular strain by the LC_50_ for the susceptible strain. The data of F_1_ reciprocal crosses between ACB-AhR and ACB-BtS were also analyzed with the equality and parallel tests using Polo Plus.

To calculate dominance, the responses of F_1_ progeny were compared with parental susceptible and resistant strains. Dominance of Cry1Ah resistance at the different toxin concentrations was measured as reported by Bourguet, *et al*.^53^ and methods adapted from Liu and Tabashnik 1997^54^. The values of degree of dominance (*h*) range from 0 that indicates completely recessive to 1 that indicates a fully completely dominant resistance and *h* value of 0.5 defines co-dominant or additive trait. The characters used in the calculation of dominance were larval survival after 7 days of infestation and the combination of the two traits.

The backcross generation obtained from mating F_1_ progenies with parental resistant strains was verified to estimate the number of genes affecting resistance by using the approach described by Tabashnik, *et al*.^10^ and using the method reported by Lande^55^. The monogenic inheritance model was tested to compare observed and expected mortality of the backcross progeny at different Cry1Ah concentrations by using the Chi-square test^10,46^. Expected mortality in the backcross can be calculated directly from experimental data following the method as described previously^32,46^. The survival percentage of larvae fed on Cry1Ah-corn and non-Bt corn was arcsine transformed to assure the normality of data prior to statistical analysis. The means difference in each corn tissue of Bt and non-Bt were tested using Fisher’s protected least significant difference (SAS Institute, Cary, NC. 2009).
